# Exploring the Impact of Ketamine on the Experience of Illusory Body Ownership

**DOI:** 10.1016/j.biopsych.2010.07.032

**Published:** 2011-01-01

**Authors:** Hannah L. Morgan, Danielle C. Turner, Philip R. Corlett, Anthony R. Absalom, Ram Adapa, Fernando S. Arana, Jennifer Pigott, Jenny Gardner, Jessica Everitt, Patrick Haggard, Paul C. Fletcher

**Affiliations:** a Department of Psychiatry, Brain Mapping Unit, University of Cambridge, Downing Site, United Kingdom; b St. John's College, Cambridge, United Kingdom; c Department of Psychiatry, Ribicoff Research Facility, Yale University, Connecticut Mental Health Center, New Haven, Connecticut; d Anaesthetic Department, University Medical Center Groningen, Groningen, Netherlands; e University Division of Anaesthesia, Addenbrooke's Hospital, Cambridge, United Kingdom; f Clinical Brain Disorders Branch, Genes, Cognition, and Psychosis Program, National Institute of Mental Health, National Institutes of Health, Bethesda, Maryland; g Cambridge University School of Clinical Medicine, Addenbrooke's Hospital, Cambridge; h University College London Medical School, London, United Kingdom; i Institute of Cognitive Neuroscience, University College London, United Kingdom

**Keywords:** Body ownership, ketamine, psychosis, rubber hand

## Abstract

**Background:**

Our sense of body ownership is profound and familiar, yet it may be misleading. In the rubber-hand illusion, synchronous tactile and visual stimulation lead to the experience that a rubber hand is actually one's own. This illusion is stronger in schizophrenia. Given the evidence that ketamine, a noncompetitive *N*-methyl-D-aspartate antagonist reproduces symptoms of schizophrenia, we sought to determine whether the rubber-hand illusion is augmented by ketamine.

**Methods:**

We studied 15 healthy volunteers in a within-subjects placebo-controlled study. All volunteers carried out two versions of the rubber-hand task, each under both placebo and ketamine infusions. In one task, they saw a rubber hand being stroked in synchrony with tactile stimulation of their real, hidden hand. In the other, stroking of the real and rubber hands was asynchronous. We recorded subjective changes in sense of ownership, as well as participants' ability to localize their hidden hand.

**Results:**

Ketamine was associated with significant increases in subjective measures of the illusion and in hand mislocalization. Although asynchronous visuotactile stimulation attenuates the strength of the illusion during both placebo and ketamine, there remained a significant illusory effect during asynchronous visuotactile stimulation under ketamine compared with placebo. The strength of the illusion during asynchronous visuotactile stimulation correlated with other subjective effects of the drug.

**Conclusions:**

Ketamine mimics the perturbed sense of body ownership seen in schizophrenia, suggesting that it produces a comparable alteration in integration of information across sensory domains and in the subjective and behavioral consequences of such integration.

Our sense of body ownership, the feeling that our body parts belong to us, although profound, is fragile. As a consequence of brain injury or mental illness, a patient may no longer feel ownership of a body part. Conversely, a person can sometimes feel that an external object is part of his or her body. Such is the case in the rubber-hand illusion (RHI) (1), in which a false sense of ownership arises from coincident but misleading visual and tactile stimuli. When one's hidden hand is stroked in synchrony with an appropriately positioned, visible rubber hand, there is a compelling experience that the rubber hand is one's own. Further, this is associated with the judgment that one's hand is closer to the rubber hand than it actually is. The power of these illusions is shown by the fact that comparable manipulations can even create a sense of being outside one's body (2–4).

The illusion usually involves a temporally precise combination of visual and tactile stimulation, although a comparable illusion can occur without visual input (5). Synchrony between vision and touch means that the participant both sees and feels a coherent sensory event, across the two sensory domains. Accordingly, factors that reduce intersensory integration attenuate the RHI. Temporal asynchrony between vision and touch is an important factor. However, other manipulations, such as positioning of the rubber hand that is clearly at odds with the position of the real hand, have a similar effect (6).

Thus, the RHI offers a way of exploring our sense of body ownership. A disturbance in this sense may be an important feature of schizophrenia (6). Indeed, the RHI is more pronounced in people with schizophrenia in that they develop the illusion more quickly and more strongly than do control subjects (7). This experience of the illusion is closely related to the intensity of symptoms—in particular, hallucinations—recorded in patients. Moreover, long latency somatosensory potentials associated with the illusion are augmented in people with schizophrenia (8). There appears, therefore, to be an alteration in the way in which sensory signals from the visual and tactile domains are integrated to produce the sense of body ownership. This may have implications for the emergence of the characteristic symptoms.

Administration of ketamine, a noncompetitive *N*-methyl-D-aspartate receptor antagonist, to healthy volunteers produces symptoms like those seen in schizophrenia (9–13). It has a marked impact on the form and ordering of thoughts (10,11,14), as well as on sensory experiences (10,13). Individuals become more sensitive to auditory information (9,12) and report changes in vision including disturbances in figure–ground relationships and in the overall salience of objects or thoughts (9). Moreover, our own experience is that volunteers receiving ketamine infusion report marked changes in bodily sensations (e.g., the sense that parts of their body were in a different position to where they actually proved to be (9,12), although these have not yet been fully or formally explored.

In this study, we sought to assess more closely ketamine's impact on sense of ownership by examining its effects on the RHI. In a double-blind, placebo-controlled crossover study we evoked the illusion in healthy volunteers. We characterized the impact of a ketamine infusion, compared with placebo, on both subjective measures of the illusion as well as a behavioral measure in which participants attempted to localize their own hidden hand. The subjective experience of the illusion was quantified using a nine-item questionnaire (1,5) (Table 1). We predicted that ketamine would increase the subjective experience of the illusion, as found in schizophrenia (7). Although the behavioral measure (hand localization) has not been explored in schizophrenia, we predicted that, on ketamine, participants would show a greater tendency to mislocalize their hand.

## Methods and Materials

### Participants

Eighteen (eight female) right-handed, healthy volunteers, with a mean age of 22.4 years, were recruited through local advertisement. The research was approved by Addenbrooke's National Health Service Trust Research Ethics Committee. All participants spoke fluent English, were nonsmokers at the time of testing, and had no history of clinical drug or alcohol abuse or of psychiatric illness. One participant was later excluded because of a subsequently discovered history of psychiatric illness, and two more were unable to complete the study because of nausea.

### The Rubber-Hand Illusion

**Figure 1:**
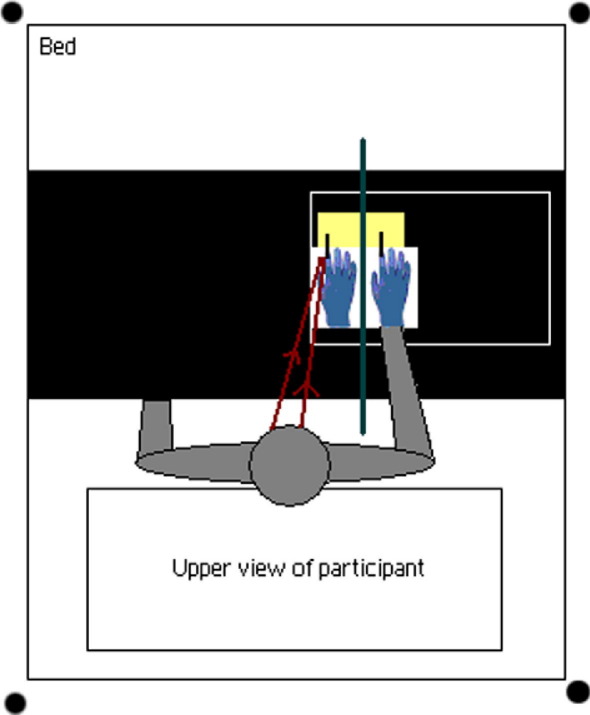
Setting of the rubber hand experiment: the participant's right hand is resting on a small table. A black cardboard box hides their hand from view, but the rubber hand can be seen by the participant (indicated by the green line). Both hands are stroked by small rotating brushes (represented by black lines) powered by a Lego motor (represented by the yellow box). The participant's right index finger is 15 cm from the index finger of the rubber hand.

The task took place at 200 ng/mL blood plasma level of ketamine, after a series of cognitive tasks run at a lower level, 100 ng/mL plasma (the data for which will be reported elsewhere). The participant's right hand rested in an open-side box (see Figure 1), the life-sized rubber hand, wearing a blue latex glove, was placed 15 cm to the left side of the participant's real hand, on which participants also wore a thin blue latex glove matching the visual appearance of the rubber hand. A black cloak was draped over their shoulder, occluding their entire right arm from view. A Lego (Billund, Denmark) motor was used to power two revolving paintbrushes, positioned to apply brushstrokes to the right index fingers of both the real and the rubber hands at approximately 1 Hz. Automated stimulation was used to ensure consistent stimulation within each session and across the placebo and ketamine sessions.

Two versions of the task were administered during a session, each lasting 5 min; in the Synchronous condition, the visible brush stroking the rubber hand rotated with the same direction, phase, and frequency as the invisible brush stroking their real hand. In the Asynchronous condition, brushes rotated in opposite directions at differing frequencies. The order of the two versions of the task was counterbalanced across sessions and participants.

### Ketamine Infusion Protocol

Bilateral intravenous catheters were inserted into the forearms: one for ketamine or placebo infusion and the other to enable serial blood sampling. In total, eight samples were taken. Racemic ketamine (2 mg/mL solutions) or saline solution were administered by a computerized target-controlled infusion (TCI) system, which calculates the infusion rates required to achieve the “target” blood concentration set by the user. Our TCI setup consists of an infusion pump (Graseby 3500, Smith's Medical, Ashford, United Kingdom), under the control of a laptop personal computer running the software Stanpump (1). Stanpump was programmed to use a two-compartmental pharmacokinetic model for ketamine (15) to calculate the infusion rates. The target blood ketamine concentration was 100 ng/mL for 60 min, and then 200 ng/mL for a further 60 min. During placebo visits, the TCI system was used in the same way, but with saline in the syringes. Each participant received both ketamine and placebo infusion on separate occasions. The order of infusion was counterbalanced but, because of participant dropout, counterbalancing was incomplete. Of the 15 participants for whom we have complete data, 9 received ketamine on their first visit. We therefore took this into account in subsequent analyses by including order effects as a covariate in the analysis of variance (ANOVA) models.

### Outcome Measures

#### Strength and Nature of the RHI

Following visuotactile stimulation, participants rated their subjective experience of the illusion using a 5-point Likert scale, in response to nine standard questions (Table 1) (1).

#### “Proprioceptive Drift.”

**Figure 2:**
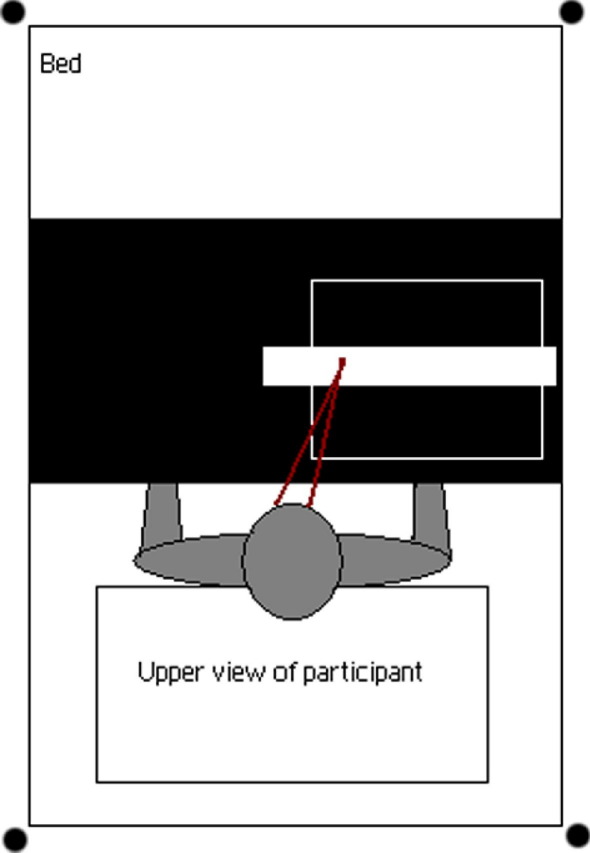
Measurement of proprioceptive drift: the participant's right hand, as well as the rubber hand, are hidden from view. A “ruler” is placed along the top of the box (represented by the white block) at predetermined, randomized, intervals. Participants are asked to imagine a vertical line from their right index finger to the ruler and to report the number corresponding to that line.

We explored the impact of the illusion on participants' estimated position of their right index finger (see Figure 2). A ruler was placed over the top of the box, and participants were asked to imagine a vertical line from their right index finger to the ruler, reporting the corresponding number at the start of the illusion and then at 1-min intervals. For each recording, both the real and the rubber hands were hidden, and the ruler was placed in a different position to avoid participants simply recalling the reading that they had given in previous measurements. The judgment recorded at each time point was normalized with respect to the initial baseline judgment, ensuring that the measure of drift reported relates to that which occurred after visuotactile stimulation had begun. Here we report the initial discrepancy between the actual and estimated positions of the real hand (before visuotactile stimulation) and the total drift (i.e., the position at the final reading relative to the estimated starting position).

### Other Effects of Ketamine

Subjective experiences induced by the drug were recorded using a series of clinician-administered questionnaires carried out by a psychiatrist. We used the Clinician-Administered Dissociative States Scales (CADSS) (16), British Psychiatric Rating Scale (BPRS) (17), Startup and Startup, and Rating Scale for Psychiatric Symptoms (RSPS) (18,19).

### Personality Measures

In addition, participants completed several questionnaires to assess personality traits. We used Eysenck Personality Questionnaire (20), Peters *et al.* Delusions Inventory (21), Chapman 1, 2, 3, and 4 (22), Multidimensional Locus of Control Scale (23), Adult Temperament Questionnaire (24), Behavioral Inhibition System/Behavioral Approach System, and the Marlow–Crowne (short) (25).

### Planned Analyses

#### Subjective Measures

First, 2 × 2 repeated-measures ANOVA was used to assess the impact of drug (ketamine vs. placebo) and task (synchronous vs. asynchronous stroking) on overall experience of the illusion, represented by an average response, of each participant, to all questions (RHI index). Post hoc paired-samples *t* tests were used to investigate these findings further.

In addition, given previous work suggesting that the first three questions in the scale are most pertinent to the illusion (1), we carried out a second analysis on responses from the first three questions only, using a 2 × 2 repeated-measures ANOVA (drug × task) including the drug-order covariate.

#### Proprioceptive Drift

The change in participant's ability to localize their right index finger, represented by “drift,” was assessed using 2 × 2 repeated-measures ANOVA. Post hoc paired-samples *t* tests were used in an attempt to discern further the individual impact of drug and task on this measure.

Finally, post hoc correlations between measures of the illusion and background personality measures as well as symptomatic effects of ketamine were carried out.

## Results

### Ketamine Levels

The mean plasma ketamine level at the time of task was 258.3 ng/mL (SD = 88.56).

### Subjective Experience of the Illusion Effects of the RHI

The RHI index (mean of all nine questions), as well as the mean response to each question (for completeness), are reported in Table 1 for each of the conditions. The statistical tests are restricted to the overall mean across all nine questions and a subsequent more focussed analysis on the key questions, 1 through 3.

### Mean of Questions 1 through 9

The effects of drug and task on RHI index (average of responses to all nine questions) were investigated using 2 × 2 repeated-measures ANOVA with order of drug administration modeled as a between-subject effect. The two factors were drug (ketamine vs. placebo) and task (synchronous vs. asynchronous stroking). A significant main effect of drug, *F*(1,13) = 9.2, *p* = .01, and of task, *F*(1,13) = 15.9, *p* = .002, was found, with the illusion proving stronger under ketamine and when stroking was synchronous. No interaction was found, *F*(1,13) = .12, *p* = .74.

In a more detailed analysis of the overall pattern of findings, paired-samples *t* tests revealed that the RHI was greater under ketamine for both synchronous (*p* = .016) and asynchronous (*p* = .019) stroking assessed separately. Moreover, synchronous stroking was associated with a greater RHI under both placebo (*p* < . 001) and ketamine (*p* = .03).

### Mean of Questions 1 through 3

In the subsequent subanalysis focusing only on mean responses to questions 1 through 3 (as described earlier), again including drug order as a between-subject factor, we observed a significant main effect of drug, *F*(1,13) = 7, *p* = .02, and of task, *F*(1,13) = 25.3, *p* < .001, with the illusion proving stronger under ketamine and when stroking was synchronous. No interaction was found, *F*(1,13) = 1.2, *p* = .3. Subsequent paired-samples *t* tests revealed that the RHI was greater under ketamine for both synchronous (*p* = .025, one-tailed) and asynchronous (*p* = .01, one-tailed) stroking assessed separately. Moreover, synchronous stroking was associated with a greater RHI under both placebo (*p* = . 000) and ketamine (*p* = .005).

In short, using both the overall subjective measure (questions 1–9) and focusing on more specific indexes (questions 1–3), there is a significant impact of ketamine during both synchronous and asynchronous stroking conditions and a significant impact of synchrony of stroking for both placebo and ketamine.

### Proprioceptive Drift

For each participant, an initial judgment, before tactile stimulation or viewing of rubber hand, was made to assess baseline judgment of hand position. The difference in this baseline judgment compared with an objective judgment made by the experimenter was assessed across placebo and ketamine sessions. The root mean square error on placebo (3.1 cm, SD = 2.3) and on ketamine (3.3 cm, SD = 2.4) did not differ significantly across the ketamine and placebo conditions, *p* = .771, paired *t* test. We can therefore be confident that drug effects were due to its impact on the illusion rather than on a general inaccuracy in hand localization. The total drift in position after 5 min, relative to each participant's estimated starting position, was entered into a 2 × 2 (drug × task) ANOVA.

**Figure 3:**
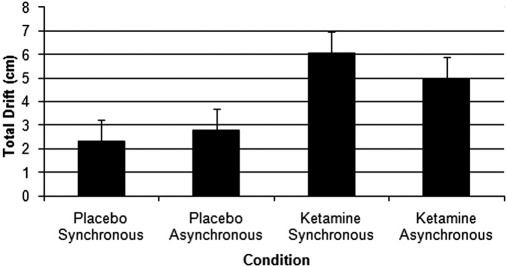
Graph to show the total drift in perceived hand position over 5 min. Error bars reflect the standard error of the drift. All four conditions are represented: temporally synchronous visuotactile stimulation on both placebo and drug and when visuotactile stimulation was temporally asynchronous for the placebo and drug conditions.

There was a significant main effect of drug on final (participant-estimated) hand position, *F*(1,14) = 5, *p* = .042, but not of synchrony, *p* = .792. Although under ketamine, the amount of subjective drift was numerically greater for the synchronous (mean total drift = 6.1 cm, SEM = 1 cm) than asynchronous (5 cm, SEM = 1.5 cm) condition, this was not a significant difference (*p* = .587). Under placebo, drift under asynchronous conditions (mean = 2.8 cm; SEM = .9 cm) was greater than synchronous (mean = 2.3 cm SEM = 1.3 cm), but did not differ significantly (*p* = .67). Finally, for synchronous conditions, drift was greater under ketamine (*p* = .039), although the same was not true for asynchronous conditions (*p* = .236). No interaction was found (*p* = .489). Results are shown in Figure 3.

### Further Exploratory Analyses

**Figure 4:**
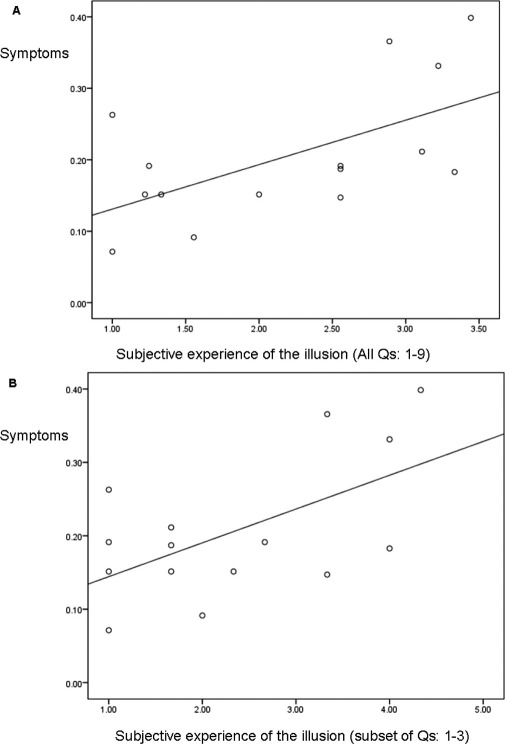
Scatter plots showing the correlation between subjective indexes of the rubber-hand illusions and symptoms induced by ketamine (combination of key questions from the Clinician Administered Dissociative States Scales and British Psychiatric Rating Scale scales). **(A)** Correlation between symptoms and overall scores on rubber-hand illusion (questions [Qs] 1–9). **(B)** Correlation between symptoms and the key subset of scores on rubber-hand illusion (questions 1–3).

We investigated whether individuals who experienced greatest changes in proprioceptive drift, might also experience the illusion more strongly as measured by the questionnaire. No significant correlation was found. We next sought to determine whether there was a relationship between RHI and symptoms produced by ketamine. No such relationship was found between effects of the RHI on hand location and a small selection of subscales rating the impact of ketamine on body perception or on psychosis-like experience. However, exploring the correlation between the subjective experience of the illusion and the symptoms induced by ketamine produced some intriguing observations. This analysis was performed separately for both synchronous and asynchronous visuotactile stimulation on ketamine. To minimize the number of comparisons, we used a composite measure of scores on key questions from BPRS (questions 6 and 7) and CADSS (2–5,8) relating to unreality and psychosis-like features. Although there was no significant correlation between symptoms and subjective experience of the illusion during synchronous stroking, there was a significant positive correlation between the impact of the drug and subjective illusory experience induced by asynchronous stroking for both the illusion as a whole (questions 1–9), Pearson *r* = .59, *p* = .02, two-tailed, and for the more constrained measure (questions 1–3), Pearson *r* = .58, *p* = .023, two-tailed (see Figure 4).

## Discussion

Healthy participants were significantly more vulnerable under ketamine to a false sense of limb ownership. The enhanced illusory effects produced by ketamine were manifest both in subjective experiences and in a greater tendency to localize the position of their real hand to that of the visible rubber hand. Note that, before the onset of visuotactile stimulation, participants were equally accurate at localizing their hidden hand under ketamine as they were under placebo. Thus, the effect of ketamine emerges following the onset of visuotactile stimulation. During ketamine administration, the experience of the illusion was enhanced even when visual and tactile inputs were asynchronous.

Under normal circumstances, body representation, and hence, presumably, sense of ownership, depend in part on multisensory integration. It has been argued that the sense of ownership inherent to the RHI is contingent on simultaneity of visual, tactile, and proprioceptive inputs (1), a conclusion given credence by observations that asynchronous visual and tactile stimulation attenuate the illusion (26–29). Moreover, the sense of ownership is associated with increased activity in multisensory areas compared with a control condition involving asynchronous stimulation (28,29). During placebo, we also observed an effect of synchronicity of visuotactile stimulation. Intriguingly, however, this was found for the subjective questionnaire measure but not the behavioral measure of drift. Furthermore, although previous work (5,30) has demonstrated correlations between proprioceptive and subjective changes associated with the illusion, it is unclear why we observed no such correlation. In one of the previous studies (5), participants were blindfolded, which perhaps increases sensitivity to proprioceptive drift by removing visual environmental cues. In the other (30), much more detailed questioning of subjective experiences enabled a factor analysis, and it was observed that proprioceptive drift was specifically correlated with one subjective component of the illusion (“embodiment”). Given that we did not establish a significant effect of synchrony on the drift measure, we are cautious in interpreting this measure here. It is worth reiterating, however, that the significant increase in drift under ketamine is not a nonspecific change in that, before the onset of visuotactile stimulation, participants were able to localize their hands as accurately under ketamine as they were under placebo.

The impact of ketamine on sense of body ownership was intriguing and in keeping with our predictions based on observations in schizophrenia. We suggest that the boost in sense of ownership is not related to an enhancement of bottom-up integrated sensory processes. If it were, the drug's effects would presumably be unique to, or greater in, the synchronous condition. Rather, it appears that the drug produces an increased tendency to accept ownership of the hand, even in the face of contradictory sensory information. One interpretation of this might simply be a nonspecific dissociative effect. However, this is unlikely given that the enhancing effect of visuotactile synchrony was preserved under ketamine. Thus, it would be wrong to conclude that ketamine attenuates sensitivity to the coherence of visuotactile information. Rather, ketamine produces an overall increase in tendency to the illusion, whereas this sensitivity to the coherence of visual and tactile input is preserved. What might be the neural mechanisms for this observed combination of effects? Two prior sets of electroencephalogram observations are relevant: first, gamma-band oscillations are enhanced in association with acute ketamine administration (31). Second, the RHI is associated with augmented gamma-band oscillations, notably under conditions of synchronous visuotactile stimulation (32,33), an effect that is not seen when stimulation is asynchronous. On the basis of these observations, we suggest that if the effect of ketamine is to augment gamma-band oscillations leading to increased cross-modal binding, this could explain the enhancement of the RHI for both synchronous and asynchronous visuotactile stimulation under drug. In particular, we suggest that during asynchronous stimulation, the ketamine-induced augmentation of gamma-band oscillation is sufficient to produce the illusion. Given this possibility, it is relevant that there was a correlation between the subjective measures of the illusion produced by asynchronous visuotactile stimulation and other measures of the subjective experience on ketamine (notably experiences of unreality assessed by combining relevant BPRS and CADSS measures). It is interesting that more spatially precise characterizations of the RHI effects in the brain have implicated a number of regions, including premotor, insula, and parietal cortices (28,34). It remains to be seen whether ketamine would boost responses during the RHI in these regions.

A further possibility is that ketamine enhances the salience of the visual input (the sight of a rubber hand in a position compatible with one's own hand) at the expense of information arising from the temporal asynchrony between sensory inputs. This would explain why the presence of the rubber hand is enough to enable the illusion to persist, albeit in attenuated form, when visuotactile inputs are asynchronous. Under normal circumstances, the appearance and orientation of the rubber hand is crucial in eliciting the illusion, and nonhand objects will not produce a sense of ownership even when stroking is synchronous, nor will a convincing facsimile of a hand produce the illusion when its orientation is clearly inconsistent with the real hand (27). Under ketamine, however, we show here that the presence of the visible rubber hand is enough to preserve the illusion even when the bottom-up signals are inconsistent. Such an imbalance in top-down–bottom-up integration may be important in understanding the psychotogenic effects of the drug (35).

How do these findings relate to schizophrenia? Peled and colleagues produced evidence that the RHI is acquired more rapidly and profoundly in people with schizophrenia than healthy control subjects (7,8), although it should be noted that their experiment did not include the asynchronous control condition and thus it is not clear whether patients would also show an increase in the strength of the illusion with asynchronous visuotactile stimulation. Moreover, they reported altered sensory-evoked potentials associated with the illusion in patients. Although we cannot tell whether the latter finding reflects a neural cause or consequence of the augmented illusion in schizophrenia, these findings are particularly intriguing in light of the fact that various symptoms of schizophrenia may reflect a disrupted sense of self (6). Passivity symptoms such as delusions of control, thought insertion, and “made” emotions seem to reflect a failure to recognize ownership of one's actions and thoughts. The experience of the self rests on a balance between bottom-up sensory percepts and top-down cognitive control. In this regard, the illusion is relevant to the disturbed sense of self in schizophrenia. Data from a number of experiments suggest that the RHI emerges from an alteration of the balance between top-down prior information about hand position, orientation, and attributes and integrated bottom-up sensations (29,34). We have previously considered the positive symptoms of schizophrenia (36) and the psychotogenic effects of drugs (35) in terms of a disturbance in this balance, such that weakened top-down signals and erratic bottom-up sensory signals can engender delusional thinking and unusual experience. The current results are, as we have shown, compatible with such a perspective.

It is also noteworthy that previous work with ketamine has produced a perturbation in right prefrontal sensitivity to mismatch signals (37), a pattern replicated in people with early symptoms of psychosis (38) and linked, in particular, to unusual thought content (37,38). A dysfunction in this region has also been suggested to be critical to delusion formation due to its role in reality evaluation (39). Moreover, a recent case study found an association between a right frontal dysfunction and anomalies in the sense of localization of self (40). Perhaps the current findings reflect a similarly localized perturbation such that a disrupted sense of ownership emerges even when visuotactile stimulation is asynchronous. Although such speculation must be cautious, our findings do highlight possible links between ketamine as a model for delusions and how the construction of the self might break down in schizophrenia. Of course, it remains to be seen whether ketamine has an impact on illusions unrelated to body ownership. Few data exist on this, but it is noteworthy that the drug has no measurable impact on the binocular depth inversion effect (41).

In summary, we have combined a pharmacologic model of schizophrenia with an illusion to which patients with schizophrenia have been shown to be especially sensitive. With ketamine, just as with schizophrenia, the illusion is enhanced. Although the precise nature of ketamine's impact is speculative, two features of its effects are noteworthy. First, it promotes an overall increase in the subjective and behavioral indexes of the illusion. Second, this effect is found even when a sensory asynchrony is present, a manipulation that would normally obliterate the illusion. Perhaps this pattern may be understood in terms of the drug's impact on the top-down–bottom-up balance that would normally account for the illusion's characteristic features.

## Figures and Tables

**Table 1:** Mean of Participant Responses to the Subjective Questionnaire

Perceptual Effects	Placebo				Ketamine			
	Synchronous		Asynchronous		Synchronous		Asynchronous	
	Mean	SD	Mean	SD	Mean	SD	Mean	SD
1. Sensation on Rubber Hand Location	4.20	1.15	1.53	1.25	4.27	1.10	2.40	1.45
2. Felt Brush on Rubber Hand	2.93	1.44	1.07	.26	3.33	1.54	1.93	1.28
3. My Hand Is Rubber Hand	2.60	1.45	1.80	1.21	3.47	1.41	2.67	1.59
4. Real Hand Drift (Toward Rubber Hand)	2.67	1.29	2.07	1.33	3.40	1.59	2.80	1.42
5. More Than One Left Hand	1.00	.00	1.13	.52	1.60	.99	1.93	1.54
6. Touch Between Two Hands (Rubber and Real)	1.87	.99	1.87	1.13	2.00	1.41	2.20	1.42
7. Real Hand Turns Rubbery	1.13	.35	1.07	.26	3.13	1.55	2.67	1.63
8. Rubber Hand Drift (Toward Real)	1.00	.00	1.00	.00	1.67	1.40	1.27	.80
9. Rubber Hand Shape and Texture Resemble Real Hand	2.47	1.51	1.80	1.32	2.73	1.53	2.00	1.60
RHI Index	2.21	.91	1.48	.81	2.84	1.39	2.21	1.42

Responses are based on a scale of 1 to 5, where 1 = “disagree completely” and 5 = agree completely. An index of effect (RHI Index) based on the mean of all responses to all questions is also represented for each condition: placebo synchronous, placebo asynchronous, ketamine synchronous, ketamine asynchronous. RHI, rubber-hand illusion.

## References

[1] Botvinick M., Cohen J. (1998). Rubber hands “feel” touch that eyes see. Nature.

[2] Ehrsson H.H. (2007). The experimental induction of out-of-body experiences. Science.

[3] Lenggenhager B., Tadi T., Metzinger T., Blanke O. (2007). Video ergo sum: Manipulating bodily self-consciousness. Science.

[4] Petkova V.I., Ehrsson H.H. (2008). If I were you: Perceptual illusion of body swapping. PLoS ONE.

[5] Ehrsson H.H., Holmes N.P., Passingham R.E. (2005). Touching a rubber hand: Feeling of body ownership is associated with activity in multisensory brain areas. J Neurosci.

[6] Waters F.A., Badcock J.C. (2008). First-rank symptoms in schizophrenia: Reexamining mechanisms of self-recognition. Schizophr Bull.

[7] Peled A., Ritsner M., Hirschmann S., Geva A.B., Modai I. (2000). Touch feel illusion in schizophrenic patients. Biol Psychiatry.

[8] Peled A., Pressman A., Geva A.B., Modai I. (2003). Somatosensory evoked potentials during a rubber-hand illusion in schizophrenia. Schizophr Res.

[9] Krystal J.H., Karper L.P., Seibyl J.P., Freeman G.K., Delaney R., Bremner J.D. (1994). Subanesthetic effects of the noncompetitive NMDA antagonist, ketamine, in humans: Psychotomimetic, perceptual, cognitive, and neuroendocrine responses. Arch Gen Psychiatry.

[10] Duncan E.J., Madonick S.H., Parwani A., Angrist B., Rajan R., Chakravorty S. (2001). Clinical and sensorimotor gating effects of ketamine in normals. Neuropsychopharmacology.

[11] Krystal J.H., Perry E.B., Gueorguieva R., Belger A., Madonick S.H., Abi-Dargham A. (2005). Comparative and interactive human psychopharmacologic effects of ketamine and amphetamine: Implications for glutamatergic and dopaminergic model psychoses and cognitive function. Arch Gen Psychiatry.

[12] Pomarol-Clotet E., Honey G.D., Murray G.K., Corlett P.R., Absalom A.R., Lee M. (2006). Psychological effects of ketamine in healthy volunteers: Phenomenological study. Br J Psychiatry.

[13] Stone J.M., Pilowsky L.S. (2006). Psychopathological consequences of ketamine. Br J Psychiatry.

[14] Malhotra A.K., Pinals D.A., Weingartner H., Sirocco K., Missar C.D., Pickar D., Breier A. (1996). NMDA receptor function and human cognition: The effects of ketamine in healthy volunteers. Neuropsychopharmacology.

[15] Absalom A.R., Lee M., Menon D.K., Sharar S.R., De Smet T., Halliday J. (2007). Predictive performance of the Domino, Hijazi, and Clements models during low-dose target-controlled ketamine infusions in healthy volunteers. Br J Anaesth.

[16] Bremner J.D., Krystal J.H., Putnam F.W., Southwick S.M., Marmar C., Charney D.S., Mazure C.M. (1998). Measurement of dissociative states with the Clinician-Administered Dissociative States Scale (CADSS). J Trauma Stress.

[17] Overall J.E., Beller S.A. (1984). The Brief Psychiatric Rating Scale (BPRS) in geropsychiatric research: I: Factor structure on an inpatient unit. J Gerontol.

[18] Chouinard G., Miller R. (1999). A rating scale for psychotic symptoms (RSPS): Part II: Subscale 2: Distraction Symptoms (Catatonia and Passivity Experiences Subscale 3: Delusions and Semi-Structured Interview (SSCI-RSPS). Schizophr Res.

[19] Chouinard G., Miller R. (1999). A rating scale for psychotic symptoms (RSPS): Part I: Theoretical Principles and Subscale 1: Perception Symptoms (Illusions and Hallucinations). Schizophr Res.

[20] Eysenck S.B.G., Eysenck H.J., Barrett P. (1985). A revised version of the psychoticism scale. Personal Individ Dif.

[21] Peters E., Joseph S., Day S., Garety P. (2004). Measuring delusional ideation: The 21-item Peters *et al.* Delusions Inventory (PDI). Schizophr Bull.

[22] Eckblad M., Chapman L.J. (1983). Magical ideation as an indicator of schizotypy. J Consult Clin Psychol.

[23] Levenson H. (1973). Multidimensional locus of control in psychiatric patients. J Consult Clin Psychol.

[24] Rothbart M.K., Ahadi S.A., Evans D.E. (2000). Temperament and personality: Origins and outcomes. J Pers Soc Psychol.

[25] Strahan R., Gerbasi K.C. (1973). Semantic style variance in personality questionnaires. J Psychol.

[26] Shimada S., Fukuda K., Hiraki K. (2009). Rubber hand illusion under delayed visual feedback. PLoS ONE.

[27] Tsakiris M., Haggard P. (2005). The rubber hand illusion revisited: Visuotactile integration and self-attribution. J Exp Psychol Hum Percept Perform.

[28] Ehrsson H.H., Spence C., Passingham R.E. (2004). That's my hand! Activity in premotor cortex reflects feeling of ownership of a limb. Science.

[29] Makin T.R., Holmes N.P., Ehrsson H.H. (2008). On the other hand: Dummy hands and peripersonal space. Behav Brain Res.

[30] Longo M.R., Schüür F., Kammers M.P., Tsakiris M., Haggard P. (2008). What is embodiment? A psychometric approach. Cognition.

[31] Hong L.E., Summerfelt A., Buchanan R.W., O'Donnell P., Thaker G.K., Weiler M.A., Lahti A.C. (2010). Gamma and delta neural oscillations and association with clinical symptoms under subanesthetic ketamine. Neuropsychopharmacology.

[32] Kanayama N., Sato A., Ohira H. (2007). Crossmodal effect with rubber hand illusion and gamma-band activity. Psychophysiology.

[33] Kanayama N., Sato A., Ohira H. (2009). The role of gamma band oscillations and synchrony on rubber hand illusion and crossmodal integration. Brain Cogn.

[34] Tsakiris M. (2010). My body in the brain: A neurocognitive model of body-ownership. Neuropsychologia.

[35] Corlett P.R., Frith C.D., Fletcher P.C. (2009). From drugs to deprivation: A Bayesian framework for understanding models of psychosis. Psychopharmacology.

[36] Fletcher P.C., Frith C.D. (2009). Perceiving is believing: A Bayesian approach to explaining the positive symptoms of schizophrenia. Nat Rev Neurosci.

[37] Corlett P.R., Honey G.D., Aitken M.R., Dickinson A., Shanks D.R., Absalom A.R. (2006). Frontal responses during learning predict vulnerability to the psychotogenic effects of ketamine: Linking cognition, brain activity, and psychosis. Arch Gen Psychiatry.

[38] Corlett P.R., Murray G.K., Honey G.D., Aitken M.R., Shanks D.R., Robbins T.W. (2007). Disrupted prediction-error signal in psychosis: Evidence for an associative account of delusions. Brain.

[39] Coltheart M. (2007). Cognitive neuropsychiatry and delusional belief. Q J Exp Psychol.

[40] Lopez C., Heydrich L., Seeck M., Blanke O. (2010). Abnormal self-location and vestibular vertigo in a patient with right frontal lobe epilepsy. Epilepsy Behav.

[41] Passie T., Karst M., Borsutzky M., Wiese B., Emrich H.M., Schneider U. (2003). Effects of different subanaesthetic doses of (S)-ketamine on psychopathology and binocular depth inversion in man. J Psychopharmacol.

